# Shifty morals

**DOI:** 10.1007/s11229-025-05088-2

**Published:** 2025-06-04

**Authors:** Aleksander Domosławski

**Affiliations:** Faculty of Philosophy, Adam Mickiewicz University, ul. Szamarzewskiego 89C, Poznań, 60-568 Poland

**Keywords:** Vagueness, Epistemicism, Inferentialism, Moral vagueness, Moral Twin Earth, Semantic plasticity

## Abstract

Epistemicism explains ignorance due to vagueness through semantic plasticity: the propensity of intensions of vague terms to shift across close linguistic communities. In the case of moral vagueness, e.g. when it’s vague whether it’s permissible to terminate a pregnancy after a certain number of days, epistemicism predicts that ‘permissible’ denotes distinct properties in different close linguistic communities. This epistemicist prediction has been pressured by arguments due to Miriam Schoenfield (Ethics 126: 257–282, 2016) as well as certain interpretations of the Moral Twin Earth cases. Schoenfield (Ethics 126: 257–282, 2016) argues that epistemicist account of moral vagueness leads to an unfeasible treatment of moral deliberation. A related worry comes from the Moral Twin Earth cases, which produce the intuition that the reference of moral terms such as ‘permissible’ remains stable across different linguistic communities. The problem for epistemicism is that metasemantic models that are meant to account for the Moral Twin Williams (Philosophical Review 127(1): 41–71, 2018) or Billy Dunaway and Tristram McPherson (Ergo 3(25): 239–279, 2016), predict that moral vocabulary is stable, which makes them incompatible with epistemicism. My aim is to make use of the inferentialist metasemantic framework presented by Robbie Williams (Philosophical Review 127(1): 41–71, 2018), and I refine it to give an epistemicist account of moral vagueness.

## Introduction

Suppose that Darryl is watching his two-year-old daughter playing in the park. Presumably it is permissible for him to look away for 5 s, but impermissible to look away for 5 min. However, we cannot point to a precise boundary between permissible and impermissible actions in this situation: we don’t know the longest period for which it is permissible to look away. Thus, ‘permissible’ seems to be a vague term.^1^

According to the epistemicist theory of vagueness, vague terms are semantically plastic, i.e. they are characterised by easy shifts in reference across close possible worlds.^2^ In Darryl’s case, the epistemicist theory predicts that we are not in a position to know the longest period that it’s permissible to look away for because ‘permissible’ could easily denote another property with a slightly different cut-off. Thus, any belief regarding the location of the cut-off is unsafe: we could easily make an error due to the shiftiness in reference of the vague term in question.

The epistemicist treatment of moral vagueness, in particular the thesis that moral terms are ‘shifty’, has recently come under pressure. Schoenfield (2016) argues that moral vagueness is incompatible with epistemicism (if we assume moral realism). The basic idea behind Schoenfield’s objections is that shiftiness in reference may be plausible for terms such as ‘tall’, but not for normatively fundamental terms such as ‘permissible’.

A related worry comes from the Moral Twin Earth cases, which produce the intuition that the reference of moral terms such as ‘permissible’ remains stable across different linguistic communities. The problem for epistemicism is that metasemantic models that are meant to account for the Moral Twin Earth intuitions, such as the models proposed by Robbie Williams (2018) or Billy Dunaway and Tristram McPherson (2016), predict that moral vocabulary is stable, which makes them incompatible with epistemicism.

The plan for the paper is as follows. Firstly, I present Schoenfield’s arguments against epistemicism. I show that the arguments rest on a certain misreading of the epistemicist theory. However, I argue that these objections pose a challenge that the epistemicist should answer. Secondly, I analyse the Moral Twin Earth cases and I show that they impose certain limitations on epistemicist metasemantics.^3^ Thirdly, I show how to adapt the inferentialist metasemantic model given by Robbie Williams (2018; 2022) to the epistemicist needs: the goal is to account for the Moral Twin Earth intuitions but keep reference of moral terms semantically plastic. I present two versions of the account that are friendly to semantic plasticity and I argue that one version has an easier task in dealing with higher-order vagueness. Lastly, I consider some residual problems for the account. I conclude that epistemicism does not have problems in accounting for moral vagueness.

## Problems for epistemicism

### Epistemicism

Epistemicism is one of the best developed views on vagueness. One of the advantages of the theory is that it does not require revisions to classical logic or the principle of bivalence. This means that the epistemicist claims that for any vague term, there is a sharp boundary delineating the things that fall in the term’s extension and those that do not. If there are such sharp boundaries, how come there is still ignorance in borderline cases?

Epistemicism attempts to explain ignorance due to vagueness by semantic plasticity:

(Semantic Plasticity) An expression is semantically plastic if and only if the shifts in the usage of language across close possible worlds imperceptibly shift the intension of that expression.

The epistemicist wants to ultimately show that semantic plasticity leads to ignorance. The use of language shifts easily across possible worlds. Intensions of words supervene on usage. Vague words are semantically plastic, so the intensions of these words shift across close possible worlds. Thus, in borderline cases a sentence such as ‘John is bald’ may be true in the actual world and false in some close possible world, due to the shift in the intension of ‘bald’. This phenomenon accounts for ignorance due to vagueness, because beliefs expressed by borderline sentences can easily be false. Thus, they are not safe, which is a prerequisite for them constituting knowledge.

Let us now attend to the problems raised for epistemicism by Schoenfield (2016).

### Schoenfield’s epistemic problem

The epistemicist treatment of moral terms has recently been challenged by Schoenfield (2016). The aim of Schoenfield’s argument is to show that the epistemicist account of moral vagueness is incompatible with moral realism. Moral realism on Schoenfield’s understanding is the claim that moral truths are necessary and part of the fundamental structure of the world.^4^ If there were a fundamental language that describes how things are on a fundamental level (such as the language proposed by Barnes (2014) or Sider (2011), then according to Schoenfield’s moral realism, moral vocabulary such as ‘permissible’ would be a part of that language. There are certain issues with this characterisation of moral realism, but we can put those aside for the purposes of the paper.^5^

Schoenfield (2016, p. 265) argues that if moral realism is true, then epistemicism cannot make sense of moral deliberation. Suppose that it is borderline whether it is permissible to terminate a pregnancy after 150 days. Cheryl is 150 days pregnant and is unsure whether it is permissible for her to terminate the pregnancy. In order to find out whether it is permissible, she consults a linguist who tells her how the word ‘permissible’ is used within her linguistic community (alternatively: she consults an anthropologist to find out what conceptual role ‘permissible’ plays within the community). On the epistemicist picture, Schoenfield claims, this would be a good way for Cheryl to proceed: whether an action counts as ‘permissible’ depends on how ‘permissible’ is used within the linguistic community. However, if moral realism is true, this is not a reasonable view of moral deliberation. To learn whether an action is permissible, one needs to find out what is true of the ‘fundamental structure of the world’: a moral question cannot be settled by learning linguistic or anthropological truths about a given community. So epistemicism has implausible consequences for moral deliberation.^6^

The argument can be summarized as follows:

1. Cheryl is in a position to know that ‘permissible’ refers to a fundamental moral property.
2. Cheryl is in a position to know the boundary for ‘permissible’.
3. Therefore, Cheryl is in a position to know facts about fundamental moral properties.

It’s worth clarifying that Schoenfield does not claim that epistemicism is committed to the view that facts about what’s permissible are dependent on facts about linguistic usage. It is possible within the epistemicist framework that ‘*A*-ing is permissible’ is false in the actual world but true in some world w* where the usage facts are different. This does not mean that *A*-ing is permissible in w*, but rather that ‘*A*-ing is permissible’ expresses a different proposition in w* that is true in w*. Therefore, Schoenfield does not claim that epistemicism is committed to moral facts being somehow dependent on facts about language or conceptual roles.

At the outset, it is worth to break down the problem raised by Schoenfield into two parts: the Epistemic Problem and the Metaphysical Problem. The Epistemic Problem is that we might come to know the answers to moral questions such as ‘is abortion after N days of pregnancy permissible?’ simply by learning what facts about (narrowly construed) usage is within a linguistic community. The Metaphysical Problem is that the fact that a linguistic community refers to a particular moral property, e.g. permissibility*, does not seem to settle the question ‘what is an agent ought to do?’, given that according to epistemicism there are multiple such properties (permissibility*, permissibility** etc.). I will focus on the Epistemic Problem in this section and I will attempt to solve the Metaphysical Problem in Sect. 5.4.

There is an established line of argumentation within epistemicism that denies the possibility of learning what the reference of a vague term is based on such a linguistic research. Since epistemicism claims that reference supervenes on usage, in order to learn what the reference of a given vague term is, one needs to know (a) what the *groundfloor facts* are (i.e. facts on which the reference supervenes) and (b) what is the function (or the semantic law) that links these groundfloor facts with facts about reference.^7^ At the outset, it is worth clarifying that the thesis that reference supervenes on usage in no way entails that it is possible to know either (a) or (b). Indeed, epistemicists like Williamson have always claimed that we should not expect such knowledge to ever be possible.^8^

Let’s first consider the issue regarding the knowability of the supervenience base for meaning. Firstly, we are not in a position to know (a), because the groundfloor facts include a broad array of facts that are not knowable by the standard linguistic methods. The notion of ‘usage’ (that intensions are to supervene on) is never very clearly specified by Williamson (1994), but he does make some remarks, which make it clear that he is thinking about usage rather broadly.^9^ The thesis that intensions supervene on usage only makes sense if we construe usage broadly. For instance, it is implausible that the meanings of natural kind terms like ‘water’ supervene on patterns of assent and dissent as demonstrated by Putnam (1975). Therefore, we should take ‘usage’ (as used by the epistemicist) to be construed broadly and serve more as a catch-all term. However, if ‘usage’ is a catch-all term that includes the impact of the external factors on the intensions of expressions, then epistemicism is not committed to the claim that the intension of ‘permissible’ is deducible from the patterns of assent and dissent. A linguist analysing patterns of assent and dissent would not be able to tell Cheryl whether abortion is permissible at 150 days of pregnancy just like he would not be able to tell her that water is H_2_O.^10^ This is important because there might be many factors that form the supervenience base for the meanings of moral terms. For instance, the permissibility of abortion in Cheryl’s case may depend on whether the foetus would survive outside of Cheryl’s body: a host of factors (the level of medical care in hospitals in Cheryl’s vicinity, the health of the foetus etc.) may have an impact here. None of these external factors would be known to the linguist (they cannot be learned simply by surveying usage). More importantly, the fact that such and such external factors play a role in determining what’s permissible might also be unknown to the linguist. A viability principle like ‘if the foetus is viable, then abortion is permissible’ is not the kind of truth one can learn by surveying linguistic usage.

Importantly, it might be the case that something like a viability principle is true despite the fact that it is rejected by all the members of a linguistic community. We may make systematic errors in our usage (Williamson, 1994, p. 207). Suppose that Cheryl is a member of a fundamentalist Christian community that all agree that destroying a foetus is impermissible after conception. By surveying patterns of assent and dissent, the linguist could say that all members of the community always assent to ‘destroying a foetus just after conception is impermissible’. They may still think that ‘impermissible’ is vague, because it is vague when exactly conception takes place. Based on the data on assent and dissent, a linguist might conclude that the boundary between permissible and impermissible destruction lies somewhere around conception (if they were trying to determine which boundary best ‘fits’ usage). However, it might still be the case that the community is just systematically wrong and the permissible/impermissible boundary lies somewhere else (e.g. around foetal viability). Thus, it is possible for a community to be systematically wrong even in non-borderline cases (if abortion is permissible until viability, then ‘destroying a foetus just after conception is impermissible’ is not only false, but definitely false). A linguist relying on patterns of assent and dissent in that community to determine the boundary for ‘permissible’ would be wrong not only about borderline cases, but also about definite cases as well.

This case shows that Schoenfield’s objection overgeneralises: it is not only an objection against epistemicism (or other *shifty views*, on which the reference of vague terms shift), but more generally against the thesis that meaning supervenes on use. Suppose that (i) abortion is permissible until viability and that (ii) foeti in the first trimester of pregnancy are clearly not viable. In such a case, there is no vagueness-related barrier to the members of the fundamentalist Christian community coming to know that first trimester abortions are permissible (their ignorance has other roots). If Schoenfield’s brilliant linguist can come to gain moral knowledge by surveying usage in borderline cases, then *a fortiori* they should be able to gain knowledge in non-borderline cases (our usage of moral terms is more stable in non-borderline cases). So they should be able to gain knowledge that first trimester abortions are permissible by surveying the usage of ‘permissible’ in the fundamentalist Christian community. But it would be weird if the linguist were able to gain moral knowledge in this way: after all, the members of the community do not know that first trimester abortions are permissible. The problem is analogous to the problem of gaining knowledge in borderline cases, but since we are talking about non-borderline cases, we are not assuming any theory of vagueness in particular: just the thesis that meaning supervenes on usage. So, it seems, Schoenfield’s argument overgeneralises: it is an argument against the thesis that meaning supervenes on use. Since it is highly probable that meaning supervenes on use, we should conclude that the problem lies with Schoenfield’s model: surveying patterns of assent and dissent is simply not a reliable method of gaining moral knowledge.^11^

One might worry that the above discussion attempts to prove to much. Surely, it is sometimes possible to gain knowledge by surveying usage. After all, at least part of the usage of moral terms like ‘permissible’ will be expressions of moral judgments. On the moral realist picture assumed by Schoenfield’s argument, it is reasonable to expect some of those judgments to be expressions of knowledge. Since it is possible to gain knowledge by testimony, it ought to be at least sometimes possible to gain knowledge by surveying people’s judgments (as any fan of *Who Wants to Be a Millionaire?* knows). However, this line of reasoning does not help Schoenfield’s case. Judgments in borderline cases are *not* expressions of knowledge: if *p* is borderline, we are not in a position to know *p*.^12^ Therefore, surveying judgments in borderline cases would not be an instance of gaining knowledge by testimony.^13^

Regarding our ignorance of the supervenience base for meaning, even more narrowly construed usage is difficult to pin down (to be of any use in learning about boundaries in borderline cases). It is not only that the reference in a public language depends on the use within the entire community (so the reference of the terms I use may change as a result of unbeknownst shifts in usage by other speakers), but also individual usage shifts with context and mood (Williamson, 1994, p. 231). Our judgments do not perfectly track the underlying phenomena, e.g. our judgments of whether someone is tall, do not perfectly track whether they possess that property (tallness), since we cannot perfectly discriminate between the tall and the not tall. Therefore, even more narrowly construed usage is somewhat messy and shifty (especially in borderline cases where our judgments are notoriously unreliable), which makes it difficult to pin down (and even more difficult to pin down with precision sufficient to be able to decide borderline cases on the basis of).

Furthermore, and crucially, epistemicists insist that we cannot know (b): the supervenience function from the groundfloor facts to facts about reference. The first thing to note is that supervenience is quite a ’tolerant’ relation: there are many ways for intensions to supervene on usage; all that’s required is that there be no change in the supervening facts without a change in the supervenience base. Therefore, there are many ways to develop a metasemantic theory that would be consistent with this supervenience claim. Epistemicists like Williamson are mostly silent about the nature of this supervenience function, because they don’t appeal to the specifics of any metasemantic theory (apart from it being consistent with semantic plasticity).^14^ Epistemicists are quite clear that the metasemantic laws in question may very well be so complex that we might never be able to generalise them sufficiently to know how they work and make specific predictions based on them (Williamson, 2004, p. 121). No linguist or anthropologist is likely to ever know such metasemantic laws to a sufficient degree that would allow them to make pronouncements in borderline cases.

Moreover, it might be that the nature of the metasemantic laws is such that they make it impossible for a linguist or an anthropologist to decide cases based on their linguistic or anthropological expertise. For instance, on Robbie Williams’s (2020) metasemantic account (that will be explored in greater detail in the subsequent sections), referents are assigned to expressions on the basis of whether they maximise the reason-responsiveness of speakers. Whether a given candidate for reference would maximise a speaker’s reason-responsiveness is not something that can be determined by linguistic or anthropological expertise.

Therefore, there are plenty of ways for epistemicism to resist Schoenfield’s challenge. At the very least, the version of epistemicism as stated by Williamson (1994) is not vulnerable to the problem; in fact, as shown above, there are arguments in Williamson’s writing already pre-emptively explaining why his theory is not vulnerable to such objections.

Of course, the epistemicist should still be careful. Since epistemicism is mostly quiet about the positive proposal for a metasemantic theory, epistemicists ought to be careful as not to propose a metasemantic theory that would be vulnerable to Schoenfield’s challenge. One version of a theory would be the crude Best Fit Model. On this model the groundfloor facts are reducible to the pattern of assent and dissent in a community, which makes the input to the supervenience function potentially knowable by a linguist. The supervenience function just assigns as reference the values that would maximise the number of utterances that would be made true by the assignment, thus potentially enabling a linguist to learn what the reference of a term is by some statistical calculation. However, there is no reason for the epistemicist to endorse such a view; in fact, as shown above, there are important considerations against adopting it.^15^

In the next section, I will consider the challenge to epistemicism posed by the Moral Twin Earth cases. I will argue that the Moral Twin Earth cases provide certain limitations on the epistemicist metasemantics for moral terms. Then, I will show how we can present epistemicist metasemantics that is not vulnerable to Schoenfield’s objections or the Moral Twin Earth cases. My goal will be to show that the epistemicist can adopt the metasemantics of moral terms presented by Robbie Williams (2018, 2020) to provide an epistemicist treatment of moral vagueness.

### Moral Twin Earth

Suppose that two communities: Kantopia and Utilitas use the term ‘permissible’ in different ways. The community on Kantopia embraces Kantian ethics and applies the term ‘permissible’ accordingly. On the other hand, the community on Utilitas are utilitarians and use ‘permissible’ in ways prescribed by utilitarianism. Suppose again that something like the Best-Fit Model holds. The reference of ‘permissible’ would be whatever property best fits with usage and maximises the number of true utterances within the community. On such a model, ‘permissible’ would have different reference on Kantopia and Utilitas since the usage is different in both communities: on Kantopia it favours the Kantian notion of permissibility and on Utilitas the utilitarian one.

Importantly, the Moral Twin Earth (MTE) case is meant to show that more sophisticated metasemantic models give us the same result. Suppose that we allow for the external-worldly facts to play a part in determining the reference of moral terms, e.g. if we allow that the property that causally regulates the use of the term ‘permissible’ is its referent. MTE cases show that this will not do, since it still seems that the properties that causally regulate the use of ‘permissible’ (and thus should be the referents of ‘permissible’ according to Putnam-style externalism) in Utilitas and Kantopia are distinct.

The Moral Twin Earth cases impose important limitations on epistemicist metasemantics. Firstly, the epistemicist metasemantics should allow for a community to believe in a false moral theory and still be able to refer to *permissibility*. Call this the Bad Theory Constraint. The Moral Twin Earth cases are meant to show that believing in an incorrect moral theory (even if that leads to an incorrect property causally regulating the usage of moral terms in a community) is insufficient to shift the reference of moral terms within a community. Kantopia and Utilitas differ with regards to the moral theories they believe in, but it does not mean that they refer to different properties when using moral terms. A community may be widely mistaken about what’s permissible and still be able to refer to the correct moral properties using their moral terms. Therefore, we want our metasemantic model to allow for communities believing in false moral theories to refer to fundamental moral properties with their moral terms like ‘permissible’.

Secondly, the epistemicist metasemantics for moral terms should allow for substantive disagreement between communities (call it the Substantive Disagreement Constraint). Horgan and Timmons (1991), in presenting the Moral Twin Earth cases, claim that there is a powerful intuition that despite the philosophical disagreement between the communities over which moral theory (Kantianism or Utilitarianism) is correct, the reference of ‘permissible’ should be the same for both communities. If ‘permissible’ on Kantopia refers to a Kantian property and ‘permissible’ on Utilitas to a utilitarian property, then when one community asserts ‘*A*-ing is permissible’ and the other asserts ‘*A*-ing is not permissible’, then both may speak truly. But if both speak truly, it seems there could be no substantive disagreement between them, even though intuitively there is. Therefore, our metasemantic model should allow for substantive disagreement between different communities if we want to accommodate the Moral Twin Earth intuitions.

At the outset let’s clarify what we might mean by ‘substantive disagreement’. First, it’s worth pointing out that the conclusions drawn from the MTE cases can be overstated at times. The Moral Twin Earth cases do not show that different communities must refer to the same moral properties. MTE cases show that our metasemantics should allow for there being a substantive disagreement between different linguistic communities. If the Moral Twin Earth cases would show that identity of reference across different linguistic communities is required for substantive disagreement, then Williamsonian epistemicism could not accommodate the conclusions from MTE. After all, epistemicism requires that different linguistic communities refer to slightly different properties when they use vague terms like ‘permissible’. Fortunately, identity of reference is not required for substantive disagreement.^16^

Suppose that the boundary for someone counting as ‘tall’ is 180 cm in height in community A and 182 cm in community B. When evaluating the height of *x*, if the speaker from A says ‘*x* is not tall’ and a speaker from B says ‘*x* is tall’, there will be a sense in which the two speakers are talking past each other. The proposition the second speaker expresses is not simply the negation of the proposition expressed by the first speaker, since the two speakers refer to slightly different properties when they use the term ‘tall’. Nevertheless, there is a strong sense that the speakers may be in substantive disagreement; there is no way for *x* to be at least 182 cm in height and for them not to be at least 180 cm in heigh. If *x* is shorter than 180 cm, the speaker from A will be wrong and the speaker from B will be right; if *x* is taller than 182 cm, the former will be right and the former wrong. Therefore, substantive disagreements between members of different linguistic communities are possible even under semantically plastic conditions.

The slight differences in assignment of referents to vague expressions are irrelevant in certain cases. However, they may become relevant in borderline cases. If a speaker from community A says ‘*y* is tall’ of a person *y* who is 181 cm in height and a speaker from B responds *‘y* is not tall’, then the disagreement is not substantive. Both speakers are speaking truly: whether *‘y* is tall’ is true depends on the assignment of an eligible candidate for the intension of ‘tall’. On some assignments, ‘*y* is tall’ comes out as true and on some as false. It seems that the two speakers in this case are not engaged in substantive disagreement.

In the moral case, we would like for substantive moral disagreement to be possible. For instance, we would like it to be the case that that at least one MTE community: Kantopia or Utilitas, has the wrong moral theory. Suppose that the Kantians are right. Then if moral realism is true, then the moral properties described by the Kantian theory are fundamental. However, since moral terms like ‘permissible’ are vague, there are multiple eligible candidates for their reference. If an act is permissible if and only if it does not violate the categorical imperative, there will be multiple candidates for the reference of ‘permissible’ that correspond to different precisifications of ‘does not violate the categorical imperative’. Since to be an eligible candidate for the reference of ‘permissible’ a property has to be fundamental, there are multiple fundamental moral properties that are the candidates for the reference of ‘permissible’.^17^ Therefore, we may allow that different linguistic communities refer to slightly different properties with their moral vocabulary; this is not a problem as long as they refer to fundamental moral properties. Since all these fundamental moral properties correspond to different precisifications of the Kantian theory, there is room for substantive disagreement. If *A* is an act that counts as ‘impermissible’ on any precisification of the Kantian theory but also maximises utility, the inhabitants of Kantopia will be right when they say ‘*A*-ing is impermissible’ and the inhabitants of Utilitas will be wrong when they say ‘*A*-ing is permissible’.

Several attempts over the recent years have been made to provide metasemantics of moral terms that would account for the Moral Twin Earth intuitions. One such prominent attempt is the account developed by Robbie Williams (2018, 2020). Williams’s original inferentialist account predicts stability of reference for moral terms across different linguistic communities. However, as I will show there are ways to tweak the account to provide a semi-stable metasemantic model that allows for both (a) a substantive disagreement between MTE communities and (b) semantic plasticity.

## Williams’s reason-responsiveness account

Robbie Williams (2018, 2020) gives an account that allows for stabilising the reference of moral terms in Moral Twin Earth cases.^18^ The account makes use of three theoretical tools: conceptual/inferential role determinism, substantive radical interpretation, and normative principles linking blame and reason-responsiveness. The three elements can be briefly outlined as follows:

*Conceptual Role Determinism*. The basic idea behind conceptual role determinism is that the reference of a term supervenes on its conceptual role within a linguistic community. Suppose that a term *W* plays the conceptual role of assigning blame: (a) when the agent judges *x*’s *A*-ing to be *W*, this makes them blame *x* for *A*-ing, (b) when the agent judges *x*’s *A*-ing not to be *W*, this prevents them from blaming *x* for *A*-ing (Williams, 2018). Necessarily, if *W* plays this role, then *W* picks out the property *P*. Let’s assume with Williams (2018, 2020) that *P* is the property of violating the categorical imperative.

*Substantive Radical Interpretation*. The radical interpretation approach to metasemantics is one that says that the correct interpretation of an agent’s utterances is the one that best explains her dispositions. On Williams’s version of radical interpretation, ‘best explanation’ is understood in terms of rationality: the correct interpretation of agent’s utterances is the one that makes them most rational. One way of cashing out this notion is via structural rationality: agent’s beliefs and desires should meet some basic structural constraints; for instance, a correct interpretation of an agent should not assign to them both the belief that *p* and the belief that *not p*. However, such structural constraints seem to be insufficient for narrowing down the correct interpretation of an agent (Williams, 2020, pp. 17–19). Therefore, Williams opts for a version of radical interpretation that supplements the structural rationality constraints with some *substantive* constraints: the correct interpretation is the one that also does not attribute to the agent some weird beliefs and desires (e.g. such as the desire for a saucer of mud^19^). Williams argues that the best interpretation of the agent’s utterances is the one that would make her the most reason-responsive.

*Normative principles*. The normative principles connect reason- responsiveness and wrongness to the effect that to be reason-responsive is to assign blame to all and only those actions that are wrong. They go as follows (Williams, 2018):

1. A reason-responsive agent would be such that: for any agent *x* and act *A*, the judgement that *x*’s *A*-ing was wrong (and unexcused), makes them blame *x* for *A*-ing.
2. A reason-responsive agent would be such that: for any *x* and *A* and for any *F* other than features that entail wrongness, the judgement that *x*’s *A*-ing is *F* would not make them blame *x* for *A*-ing.
3. A reason-responsive agent would be such that: for any *x* and *A*, the judgement that *x*’s *A*-ing is not wrong, would make them refrain from blaming *x* for *A*-ing.
4. A reason-responsive agent would be such that: for any *x* and *A* and for any *G* other than wrongness entails, a judgement that *x*’s *A*-ing is not *G* would not make them refrain from blaming *x* for *A*-ing.

These three elements combined allow Williams to have semantic stability across different linguistic communities. In the Moral Twin Earth case, if wrongness is the property of violating the categorical imperative, then even for the consequentialist community their term ‘wrong’ refers to wrongness, because ‘wrongness’ plays the same conceptual role in the two communities and only under this interpretation we maximise their reason-responsiveness: only the fact that some action is wrong gives us a reason to blame someone for performing it.

As briefly pointed out in Sect. 2.2, there is no way for a linguist to learn (using standard linguistic or anthropological methods) about the reference of moral terms like ‘wrong’ or ‘permissible’ simply by surveying usage (or the conceptual roles) within a linguistic community. That is because to know (using such a linguistic analysis) what a moral term refers to would require the knowledge of what makes an agent most reason-responsive in their use of moral terms. However, that would require substantive ethical knowledge (such as the knowledge that wrongness is the property of violating the categorical imperative). So Williams’s account (and my epistemicist-friendly extensions that also endorse the reason-responsiveness constraint) are not vulnerable to Schoenfield’s Epistemic Challenge.

Therefore, Williams’s account gives us stability of moral terms and allows for the possibility of systematic error. Thus, it satisfies the Bad Theory constraint discussed in the previous section and reliably produces the result that moral terms refer to fundamental moral properties (since they are the ones that would make the communities most rational if we assign them as referents to their moral terms). However, as it stands the account does not seem to allow for semantic plasticity: it forces absolute stability of reference without sensitivity as to whether the terms in question are stable or plastic. The task in the next section will be to refine the framework to accommodate semantically plastic moral terms. This will allow us to give an epistemicist treatment of moral vagueness in later sections.

## The epistemicist-friendly tweak

I can see two ways to adjust the reason-responsiveness approach to make it more flexible and allow it to deal with vagueness. I will call them Solution A and Solution B. I think both approaches are initially promising. However, I think Solution A faces a serious problem when it comes to dealing with higher-order vagueness. Solution B is much better equipped for this task. Therefore, I will opt for Solution B in the end.

### Solution A

Solution A^20^ relies on relaxing the Conceptual Role Determinism thesis and the four normative principles so that multiple candidates are allowed as referents of ‘wrong’. The philosophical justification for this move is that in borderline cases it does not seem like assigning one as opposed to another candidate as the reference of one’s utterances makes one more reason-responsive. In order to do this, we have to firstly relax Conceptual Role Determinism. Instead of allowing for there being only one candidate for the reference of *W* for any given conceptual role *R*, we allow that there are multiple candidates (call them wrongness-candidates) for the reference of *W*. The idea is that an interpretation of *W* as referring to one of the wrongness-candidates does not make an agent any more reason responsive than an interpretation of *W* as referring to another wrongness-candidate: multiple interpretations make the agent equally reason responsive.

Secondly, we have to adjust the normative principles, so that they allow for multiple wrongness-candidates. Let’s say that an act is determinately wrong (determinately not wrong) if it is wrong (not wrong) according to every wrongness-candidate. Our relaxed normative principles are given by:

1. A reason-responsive agent would be such that: for any agent *x* and act *A*, the judgement that *x*’s *A*-ing was determinately wrong (and unexcused), makes them blame *x* for *A*-ing.
2. A reason-responsive agent would be such that: for any *x* and *A* and for any *F* (such that it is not the case that *F* entails any of the wrongness-candidates), the judgement that *x*’s *A*-ing is *F* would not make them blame *x* for *A*-ing.
3. A reason-responsive agent would be such that: for any *x* and *A*, the judgement that *x*’s *A*-ing is determinately not wrong, would make them refrain from blaming *x* for *A*-ing.
4. A reason-responsive agent would be such that: for any *x* and *A* and for any *G* (such that *G* is not entailed by every wrongness-candidate), a judgement that *x*’s *A*-ing is not *G* would not make them refrain from blaming *x* for *A*-ing.

Importantly, what the revised principles tell us is that if an act is definitely wrong, then a rational agent would blame one for committing it and if an act is definitely not wrong then a rational agent would not blame one for performing it. But the principles give us leeway in borderline cases: we are neither required nor forbidden by the normative principles to blame someone for performing a borderline wrong act. On such an approach the rationality considerations are constraints on reference, but don’t uniquely secure reference if there are multiple candidates for the reference of ‘wrong’ (i.e. if there is more than one property among the *P*s).

The refined principles have an advantage over the initial version, because they don’t force precision where it is not required. One problem that arises though has to do with the opposite worry that the principles may produce vagueness where we require precision. For one thing, it would be desirable to have metasemantics that would be capable of handling both vague and precise vocabulary. Fortunately, the refined principles don’t require vagueness nor precision: the principles work equally well when there is one or multiple candidates for the reference of a term (in our case ‘wrongness’); in case there is only one admissible candidate, the refined principles are equivalent to the unrefined ones.

Here is the picture so far. For the two Twin Earth communities the use of the term ‘wrong’ plays the same coarse-grained conceptual role. This means that despite different beliefs about the subject matter (what is wrong, what is permissible) the reference of the crucial terms like ‘permissible’ or ‘wrong’ is roughly the same for both communities. This means, for instance, that if Kantianism is true, both communities are interpreted as referring to a Kantian property. This much we get from the refined Williamsian metasemantics. However, the relaxed principles allow for multiple candidates for the reference of moral terms like ‘wrong’: they impose constraints on reference, but don’t force the stability of reference. Williams’s model allows us to get the desired semi-stability. There remains a matter of accounting for the semantic plasticity of moral terms. Epistemicism requires that different close linguistic communities refer to slightly different properties in case of vague terms. Therefore, we can let usage decide which particular candidate (among the ones that satisfy the refined normative principles) is assigned as the reference of moral terms for a given linguistic community.^21^

Unfortunately, one important problem arises for the account: higher-order vagueness. With Solution A we managed to explain first-order moral vagueness (i.e. vagueness as to where the permissibility-impermissibility boundary lies): there are multiple candidates for the reference of ‘permissible’ that are admissible by the lights of our normative principles and no candidate is the definite referent of ‘permissible’. However, using Solution A we cannot explain why we don’t know where the second order boundary (e.g. the boundary between definitely permissible acts and borderline permissible acts) lies. On the standard epistemicist model, higher-order vagueness is explained by intransitivity of the accessibility: the set of admissible precisifications changes across nearby possible worlds as the supervenience base for meaning shifts. However, this tactic is unavailable on Solution A, where the set of admissible precisifications is determined by the coarse-grained conceptual role of ‘permissible’. Since this role is coarse-grained, in any nearby world, the coarse-grained conceptual role is the same as in the actual world. Consequently, there the set of admissible precisifications in any nearby world is the same as in the actual world, so we don’t get the desired intransitivity (all the precisifications that are actually admissible, are also admissible in every nearby world). In order to get intransitivity, we would have to allow for shifts in the coarse-grained conceptual role across nearby worlds. However, if we have to rely on the shifts in the conceptual roles across nearby worlds to explain higher-order vagueness, it would be more elegant to rely on this explanation for the case of first-order vagueness for the sake of the uniformity of explanation. This is the path taken by Solution B.

### Solution B

Fortunately, there is a way to give an epistemicist account of higher-order vagueness within this framework. Solution A requires adjusting the original principles proposed by Williams. On the other hand, Solution B requires slightly changing the notion of *conceptual role*, while leaving the rest of the framework intact. As so far described, the conceptual roles have been quite coarse-grained: many patterns of behaviour and inference have qualified as being the same conceptual role. Solution B relies on the idea that conceptual roles are taken to be more fine-grained: a narrower range of behavioural patterns counts as constituting the same conceptual role. The notion of a conceptual role is not a precise one: there is no precise boundary between communities that use the term ‘wrong’ in the same way that we do. On Williams’s account, ‘wrongness’ plays the *blame* assignment role in our community. However, it is vague what constitutes blame; for instance, it is unclear how strong a reactive response must be to constitute blame. Communities that differ slightly with the strength of the reactive response (e.g. in one community it is blame*, in another it is blame**), will differ slightly with regards to the conceptual role that ‘wrongness’ plays: in one community it will be the blame* assignment role and in the other the blame** assignment role.^22^

The heart of Solution B is this: small shifts in conceptual role across close possible worlds produce small shifts in the reference of moral terms that explain the vagueness in a standard way. On the fine-grained construal of conceptual roles, communities in nearby worlds will differ slightly with respect to the conceptual role that ‘wrongness’ plays. Conceptual Role Determinism holds for ‘wrongness’ in all nearby worlds– identity in conceptual role entails the same intension for ‘wrongness’. Williams’s normative principles dictate which property is denoted by ‘wrongness’ in each community: in a community where ‘wrongness’ plays the blame* assignment role ‘wrongness’ denotes wrongness*, in a community where ‘wrongness’ plays the blame** assignment role, ‘wrongness’ denotes wrongness** etc.

The solution to the initial puzzle then is this. Small shifts in the conceptual role of ‘wrongness’ across close possible worlds shift the reference of ‘wrongness’. In a borderline case C, it is indefinite whether for some act *A* committed by *x*, *A*-ing is wrong. The epistemicist explanation of vagueness is that the reason why C is a borderline case is that ‘wrong’ denotes different properties in distinct communities. For instance, it denotes wrongness* in w* and wrongness** in w** that is close to w* and *A*-ing is wrong* but not wrong**. Suppose that blame* and blame** are reactive responses differing slightly in strength (blame* is slightly weaker than blame**). Suppose that in w* ‘wrong’ plays the blame* assignment conceptual role and in w** it plays the blame** assignment conceptual role. Then, a reason-responsive agent would be such that they would blame* *x* for *A*-ing but would not blame** *x* for *A*-ing (we can rephrase Williams’s four normative principle using the notions of wrongness*/blame* and wrongness**/blame** respectively).

Solution B allows us to account for higher-order vagueness on this account. Small shifts in the conceptual role of ‘wrong’ shift the reference across the modal space. Once the shifts in the conceptual role of ‘wrong’ add up in some world *v*, it no longer counts as being relevantly close to the actual world. We are not in a position to know where the cut-off point between ‘definitely wrong’ and ‘borderline wrong’ is, because there are nearby (relevantly indistinguishable) worlds that *v* is close to (even though it is not close to the actual world). Thus, the same explanation (small shifts in conceptual role shifting the reference slightly) applies to vagueness at different orders.

Now that we have the above metasemantics borrowed (and adapted) from Williams, we can try to go back to the initial objections posed by Schoenfield (2016) to explain why, despite their initial plausibility, the objections don’t work. Schoenfield’s objections presupposed a simple epistemicist model, on which it seems it would be possible to learn what moral facts are from the facts about usage. The epistemicist can object that we don’t know what the usage facts are and how these facts alongside the metasemantic laws determine the meaning facts. However, this might not convince critics like Schoenfield, who can insist that even though we don’t in fact know the usage facts or the metasemantic laws, in principle that might be possible (especially if we adopt an idealisation that it would be in principle knowable for an omniscient anthropologist or linguist).

Nevertheless, we can answer the objections more directly using the new metasemantics. On the metasemantics presented above, it is not possible to learn moral facts without engaging in moral deliberation (Williams, 2018). It is not enough to know how the metasemantic rules determine the reference of moral expressions. Different moral theories will disagree over which acts are to be categorised as morally wrong. The disagreement over the first order moral theory will seep into the metasemantics of moral terms. The reference of moral terms is determined by which moral theory is correct, because only interpreting agents as referring to *wrongness* (when using the term ‘wrong’) would make them most reason-responsive. Determining which property that is, is the task of the first order moral theory.

Therefore, Cheryl will not be able to learn after which *N* days abortion becomes impermissible after talking to an idealised anthropologist (unless the anthropologist has consulted some experts on morality who have established what the true moral theory is). Nevertheless, we have a way of accounting for vagueness as outlined above. Hence, by adopting the framework from Williams (2018) we have a semi-stable metasemantic theory for moral terms that is compatible with epistemicism while satisfying the demands presented by Schoenfield (2016) objections. The theory also satisfies the constraints presented by the Moral Twin Earth cases. In the next section, I will consider potential problems for the account, and I will argue that these issues can be dealt with.

## Potential problems

### Fine-grained conceptual roles

Solution B relies on fine-grained conceptual roles. The difference between the two proposals should be framed as a difference between modally plastic and modally robust properties (Dorr & Hawthorne, 2014). For instance, the relation *λxλy. x is within the eyesight of y* is a modally robust relation: we could significantly change the distance between *x* and *y* and the relation would still hold (in many cases). On the other hand, the relation *λxλy. x is exactly the same height as y* is not modally robust: changing the height of *x* even slightly means that it stops being exactly the same height as *y*. If the relation *λxλy. x has the same conceptual role as y* is construed as a modally plastic relation, then we can easily give an account of moral vagueness using Solution B. On such a construal it is highly contingent what conceptual role a given moral term plays within a community. We can then use the original Williamsian normative principles and Conceptual Role Determinism to explain vagueness of moral terms: slight shifts in conceptual role of moral terms slightly shift the reference of moral terms across linguistic communities. This gives us a uniform explanation of moral vagueness at every order.

One might worry that the construal of conceptual roles as fine-grained is unprincipled. But there is philosophical justification for this. Suppose that there is some far away possible world w* where the conceptual role of ‘wrongness’ is clearly distinct from the role it plays in our community. We assumed that the conceptual role of ‘wrongness’ is defined by its relationship to *blame*: all and only acts that are wrong deserve blame. When someone commits a wrong act, we blame them, which means that we hold some negative feelings towards the perpetrator. In case of horrifically wrong acts, such as e.g. starting an unjust war, our feelings towards the perpetrator are quite strong. Suppose that at w* the community’s feelings towards perpetrators of horrific acts such as starting an unjust war are very mild, comparable to the negative feelings we might feel towards someone wearing unmatching socks. I think it is clear that the very slight negative emotion they feel towards horrific acts is not blame, it is something clearly distinct (say blame*). Their use of the term ‘wrong’ is associated with the link to blame* and not with blame– blame requires feelings of appropriate strength. So ‘wrong’ plays a very different conceptual role in the actual world and at w* and refers to a different property– not *wrongness* but some other property, e.g. wrongness*. If this is the case, then there is a series of worlds between w* and the actual world, where the strength of the negative emotion towards wrong acts is slightly larger with every consecutive world. In such a case, it is natural to suppose that the conceptual role of ‘wrong’ in each world is slightly different, which produces small shifts in the reference of ‘wrong’ in each world. Of course, it is not inconsistent to suppose that conceptual roles are modally robust; however, such a construal is less natural in the current setting.

In discussing Ralph Wedgwood’s (2001) view, on which it is also the case that the conceptual role a moral term plays determines its reference, Schoenfield (2016, p. 273) worries that:

For if the vagueness that arises in Amputations [a case of moral vagueness] were a result of indeterminacy about which rules constituted the conceptual role for “better than,” then fixing what the rules are would fix which precise relation is the referent of “better than.” But this is implausible. Fixing the rules of practical reasoning that define the conceptual role for “better than” (say we use the subjective notion) doesn’t determine a cutoff in the Sorites series for Amputations.

But it is not clear why Schoenfield takes the view to be implausible. If we fix the conceptual role a moral term plays within a community (e.g. by specifying the intensity of blame the community uses), then we would fix the boundary of that term. This does not mean that we would be able to come to know what the reference of moral terms, but merely that there is a unique property such that when assigned as the reference for a moral term, it maximises the reason-responsiveness of the speakers within a community.

### Vagueness in the levels of blame

Just as it is vague which actions are blameworthy, it is also vague how much blame a given act deserves. Suppose that we can order levels of blame by the intensity of negative emotion, e.g. so that the level of blame *N* corresponds to giving someone a dirty look for 5 s etc. Consider the following sentence:

(S1) Darryl’s looking away for 200 s, makes it appropriate to treat him with a level of blame *N*.

The worry is that Solution B may make sentences like (S1) have a definite truth value, even though they are intuitively vague.

Whether (S1) is vague will depend on the *structure* of moral properties. Suppose that a version of the *seriousness gradualist* (Hurka, 2019) picture is correct: some wrong acts are worse than others, e.g. murder is worse than a traffic violation. Consequently, worse acts deserve higher levels of blame. Under gradualism, (S1) will have a definite truth value if the assignment of infractions to levels of blame is stable across the nearby linguistic communities, i.e. all the communities will agree that Darryl’s looking away for 200 s, makes it appropriate to treat him with a level of blame *N* etc. However, we do not have to go for this kind of picture.

Firstly, we may reject gradualism. One reason to do this is the idea that the difference between permissible and impermissible actions is a very significant one; there is no smooth transition between permissible and impermissible actions in the levels of blame we assign to these actions. Acting impermissibly deserves blame; acting permissibly deserves *no* blame. Suppose that Community A draws the permissible/impermissible boundary at 300 s and Community B draws it at 200 s. If someone looks away for 250 s, Community A will deem the action ‘permissible’; if we reject gradualism, looking away for 250 s merits no blame according to Community A, but does according to B. So Community A and Community B will disagree over the appropriate level of blame and sentences like S1 will be vague.^23^

Secondly, we may reject the stability of the gradualist mapping from infractions to levels of blame. It might be that Community A and Community B disagree over the appropriate level of blame for looking away for 200 s, e.g. in the dialect of Community A, ‘level *N* of blame is appropriate’ is true and in the dialect of Community B, ‘level *M* of blame is appropriate’ is true (with *N ≠ M*). Therefore, Solution B would make sentences like S1 have a definite truth value only if we adopt additional controversial assumptions.

### Metaethical implications

A potential worry for the epistemicist account outlined above is that it implicitly relies on a specific metaethical theory. In presentation of Solution B, I relied on the idea that communities in nearby worlds differ with respect to the conceptual role played by ‘wrongness’. Crucially, I also relied on the idea that Williams’s normative principles will select different properties as referents of ‘wrongness’ in communities where the conceptual role is slightly different, i.e. that the normative principle tells us that wrongness* is the appropriate referent for a community where ‘wrongness’ plays the blame* assignment role and so on for other variants of blame. One might worry that this kind of picture might not be compatible with every kind of metaethical theory out there.

First, looking at the dialectical situation, the goal for the epistemicist is to resist the challenge as presented by Schoenfield (2016) that epistemicism is incompatible with moral vagueness, given moral realism. We have operated under the assumption that moral realism is true: that there really are fundamental moral properties. If the view outlined above is right, then moral realism is compatible with epistemicism. There might be challenges to epistemicism from different metaethical views and there might be versions of moral realism that take on additional assumptions that would make them incompatible with epistemicism. However, we have shown that epistemicism is compatible with a broad realist picture of the moral realm.

Second, it is not clear that any challenge to epistemicism from metaethical consideration will not just be a challenge to moral vagueness. For instance, some metaethicists might be unhappy about the fact that epistemicists predict that some moral truths are unknowable.^24^ However, many theories of vagueness predict that if $\:\varphi\:$ is borderline, then $\:\varphi\:$ is unknowable; such metaethicists should just deny that there is moral vagueness and claim that our ignorance in problematic cases (like Darryl’s) is just a result of us not knowing enough about morality. Some metaethicists are also unhappy with the result that small changes in the underlying physical facts (such as the amount of time Darryl spends looking away from his daughter) shift the action from a permissible to an impermissible territory.^25^ However, this is a qualm about classical logic and bivalence rather than the epistemic view of vagueness specifically (Hawthorne, 2022, p. 229). Vagueness and the Sorites simply sometimes have surprising consequences.

Some metaethicists may also be unhappy about the fact that epistemicists predict that there are multiple fundamental moral properties that are candidates for the reference of moral terms; however, again, the vast majority of theories of vagueness (including ontic theories of vagueness^26^ supported by Schoenfield) predict that for any vague term there are multiple eligible candidates for its reference (I expand on that point in the next section). So, there is no obvious challenge to the epistemicist that would not also be a challenge to moral vagueness in general.

### Schoenfield’s metaphysical problem

Recall that I have divided the problem raised by Schoenfield into two issues: the Epistemic Problem and the Metaphysical Problem. The Epistemic Problem for the epistemicist to solve was to show that we cannot come to know the answers to moral questions like ‘is *A*-ing permissible?’ by surveying data about linguistic usage (or the role that moral terms play in a linguistic community). This objection was dealt with in Sect. 2.2.

Schoenfield (2016, pp. 269–271) worries that a metasemantic model, on which we cannot infer facts about the boundary for ‘permissible’ from surveying usage, will entail that the epistemicist is committed to ontic vagueness. The reason is that on the epistemicist view, it should be possible to precisify terms by agreeing on usage so that certain candidates for reference are excluded. For instance, if we all assented to ‘it’s impermissible for Darryl to divert attention for 40 seconds’, then this sentence would be definitely true. If the candidate for the reference of ‘permissible’ on which ‘diverting attention for 40 seconds is permissible’ is true remains a candidate for the reference of ‘permissible’ (even if we all agreed to exclude it), the vagueness is ontic. Part of the response to this objection is that on the model presented above, the pattern of assent and dissent of speakers does not have an impact on the reference of moral terms as envisioned by Schoenfield. In terminology introduced by Sud (2019), the pattern of assent and dissent is *impotent*: it is the conceptual role that determines the reference of moral terms.

However, Schoenfield is still worried that epistemicism is committed to multiple moral properties. This is what I dubbed the Metaphysical Problem: the fact that a linguistic community refers to a particular moral property, e.g. permissibility*, does not seem to settle the question ‘what is an agent ought to do?’, given that according to epistemicism there are multiple such properties: permissibility*, permissibility** etc. (Schoenfield, 2016, p. 273). If there is a borderline case such that it is permissible* to *A* but it is impermissible** to *A*, it seems that there remains a question: ‘what ought a rational agent do?’.

Suppose that we accept that there are multiple fundamental moral properties with associated notions of ‘ought’, ‘permissible’ etc. This means that in borderline cases questions like ‘what ought I *morally* do?’ will not have definite answers. The further question that seems to be unanswered in a borderline case even then is ‘what ought I *practically* do?’. Practically, is it permissible for me to *A* only if all the permissibility-like properties agree that ‘*A*-ing is permissible’ or can I *A* just in case at least one such property classifies *A*-ing as ‘permissible’? Decision making under determinacy is a topic that’s gaining more attention recently (e.g. Williams, 2014, 2017; Peterson, 2023, Ch.7). I do not have the space to discuss all the options; it would take a separate paper to discuss all the issues in sufficient depth.

One point that is worth making here, however, is that decision making under indeterminacy is a problem independent of epistemicism. Rather it is an issue that every theorist of vagueness must face. Moral vagueness commits us to the thesis that there are multiple equally fundamental candidates for the reference of ‘permissible’. The ontic interpretation of moral vagueness proposed by Schoenfield (2016) does not fare any better here. For proponents of the ontic interpretation of moral vagueness, it is indeterminate what one ought to do in a borderline case. If it is indeterminate whether one ought to *A* (or as some put it ‘there is no fact of the matter’ as to what one ought to do) then we also have an action guiding problem: what ought one do if it is indeterminate whether one ought to *A*? For the ontic vagueness theorist, the vague property *permissibility* has multiple eligible cut-off points; for the epistemicist, there are multiple interpretations of ‘permissible’ in different linguistic communities that denote different properties (permissibility*, permissibility** etc.). Any theory of vagueness will have to be supplemented with a theory of decision making and action under indeterminacy.^27^ However, that would be an entirely different project; for now, it does not seem like epistemicism in particular is faced with a comparatively more difficult task here.

The ontic vagueness theorist may attempt to underline the difference between the two theories. The ontic theory claims that there is only one moral property (in the vicinity), namely *permissibility*, whereas epistemicism claims that there are multiple such properties. However, the difference here between the two theories is immaterial. Both theories need the claim that there are multiple eligible candidates for the reference of ‘permissible’. Moreover, it cannot be a fundamental fact that one of the candidates is more eligible for reference (then the reference would be determinate). Where there is a difference between the ontic and the epistemic view is in communication. On the ontic view, different linguistic communities are assigned the same interpretation, so there are no issues with miscommunication resulting from the difference in interpretations. On the other hand, on the epistemic view different linguistic communities are assigned different interpretations, which potentially results in miscommunication. However, that is a well-known feature of the epistemicist account and is by no means limited to the moral case. Therefore, moral vagueness does not present any special problems here.

Lastly, accepting that there are multiple eligible ‘oughts’ (or multiple eligible cut-offs for ‘permissible’) may seem like a steep price to pay. However, it’s a price to be paid by anyone who believes that there is moral vagueness: if $\:\varphi\:$ is vague then there are multiple candidates for its reference. This includes non-epistemic theories. If one cannot bear to pay the price of multiple candidates for ‘permissibility’, one ought to deny that there is moral vagueness.

## Conclusion

In this paper I presented an epistemicist-friendly, semi-stable inferentialist metasemantic theory of moral terms. First, I unpacked Schoenfield’s argument against semantically plastic moral terms. I argued that upon reflection on the epistemicist metasemantic commitments, most of the objection’s thrust is blunted. I also showed that the Moral Twin Earth cases impose certain constraints on the epistemicist. However, I argue that the epistemicist should provide a model that would alleviate any worries regarding its compatibility with moral vagueness and the lessons from Moral Twin Earth. Consequently, I used Robbie Williams’s (2018, 2020) inferentialist metasemantic framework to provide such a model. I presented two ways of combining Williams’s framework with epistemicism and I argued that one has an easier way with dealing with higher order vagueness. In the last section, I argued against potential challenges for the account. When the dust settles, it turns out that for all we know moral vagueness just is epistemic vagueness.

## Data Availability

Not applicable.

## Footnotes

1 There are ways to resist the claim that cases like this one entail that ‘permissible’ is vague (see Magidor (forthcoming)). Perhaps the reason for our ignorance is that we simply don’t know enough about all the factors that we would have to take into account to determine whether an action is permissible. However, for the purposes of this paper I assume that examples like this are instances of moral vagueness.

2 This version of epistemicism which explains vagueness via semantic plasticity has been developed by Williamson (1994). For other versions of epistemicism see Breckenridge and Magidor (2012) or Sorensen (1988). For the purposes of this paper, whenever I use the term ‘epistemicism’ I mean the Williamsonian version of the theory.

3 See Manley (n.d.) for an epistemicist account of moral vagueness that rejects the Moral Twin Earth intuitions.

4 Schoenfield follows the characterization of moral realism from Dougherty (2014).

5 For some problems with the characterisation of moral truths as necessary truths see Hawthorne (2022). Hawthorne (2022, p. 222) argues that the modal status of moral truths is a red herring. For instance, even if it is impermissible for Darryl to look away from watching his daughter for 5 min, surely this is not necessarily so. It would be permissible for him to look away if he knew that his daughter would be tortured if he does not look away.

6 Schoenfield (2016, p. 262, footnote 12) claims that the problem for epistemicism will not arise on some first-order ethical theories. Scalar Utilitarianism (Norcross, 2006) takes ‘better than’ and not ‘permissible’ to be a fundamental moral term. Therefore, since ‘permissible’ is not a fundamental term, any account of the vagueness of ‘permissible’ will not be in tension with moral realism. I believe that Schoenfield is mistaken here: it is also vague which actions are better or worse. For instance, we might reasonably ask whether in Darryl’s case it is at any time better for him to look away (e.g. thereby maximising his pleasure by reading a newspaper) or to pay attention to his daughter. Nevertheless, this problem can be neglected for our purposes here since if I am right, then the epistemicist has more problems to solve.

7 The terminology of ‘groundfloor facts’ comes from Hawthorne (2006).

8 See Williamson (1994, §7.5; 1997a, pp. 221–222; 1997b, p. 258; 2004, p. 121) and Hawthorne (2006, pp. 199–201).

9 Williamson (1994, p. 205) states: ‘environmental factors beyond our dispositions to assent and dissent may play a role in fixing truth-conditions’.

10 I simplify a bit here: a current linguist could potentially discover the fact that water is H_2_O simply by analysing the patterns of assent and dissent, because this is a commonly known fact the statement of which would be assented to by many speakers. The wider point stands about a linguist living before the discovery of the chemical structure of water: they would not be able to deduce that water is H_2_O from the patterns of assent and dissent.

11 In Williamson’s (1994, p. 206) words: ‘Although meaning may supervene on use, there is no algorithm for calculating the former from the latter. Truth-conditions cannot be reduced to the statistics of assent and dissent.’

12 Borderlineness incompatibility with knowledge is accepted by most theories of vagueness (including epistemicism). For a dissenting view, see Dorr (2003).

13 Similarly, members of the fundamentalist Christian community do not know that first term abortions are permissible (because they don’t believe it), so they cannot transfer their knowledge to the surveyor.

14 In his reply to Schiffer, Williamson (1999, p. 511) notes: ‘epistemicism is consistent with many different more or less vague theories of reference; for example, it does not dictate the relative weight of causal and descriptive considerations in determining reference. Of course, not everything goes. Epistemicism is inconsistent with extreme verificationist theories of reference. But neither Schiffer nor anyone else has shown epistemicism to be inconsistent with any independently plausible theory of reference. My conjecture is that any theory of reference incompatible with epistemicism will also be objectionable on grounds independent of vagueness.’

15 There is also a worry that even if the epistemicist were to adopt the crude Best Fit Model, it would not allow us to resolve moral dilemmas, because the Best Fit Model undermines (1), i.e. the knowledge that moral terms pick out fundamental moral properties. If the Best Fit Model were correct, then learning what ‘permissible’ refers to would not be a reliable way of learning fundamental moral truths, because there is no guarantee that ‘permissible’ refers to a fundamental moral property on that model. If we assume the safety conception of knowledge (assumed by epistemicism), this means that surveying usage would not be a safe way of generating beliefs about what’s permissible, so would not count as knowledge. Thus, we would not be able to come to know the answers about fundamental moral facts even on the Best Fit Model.

16 This is also a point powerfully made by Plunket & Sundell (2013), who take Moral Twin Earth cases to be instances of *metalinguistic negotiation*. Two speakers who utter ‘*A*-ing is permissible’ and ‘it’s not the case that *A*-ing is permissible’ may refer to different properties when they use ‘permissible’, but their tacit disagreement is over which property should be used in this context. The problem with Plunkett & Sundell’s (2013) view (one they acknowledge) is that their solution for the Moral Twin Earth problem does not seem to work for *basic* normative terms, i.e. terms that play a role in evaluating which term *should* be used in a given context. Since we want a solution that would also be able to handle such basic normative terms, we have to go another way.

17 Note that the claim that vagueness requires that there be multiple candidates for reference is not just the requirement of epistemicism. Other theories of vagueness, e.g. semantic or ontic, also have this requirement.

18 Robbie Williams (2018, 2020) presents his account as a theory of mental representation; I will focus on linguistic representation, but I am not distinguishing the two for the purposes of this paper (if a linguistically competent agent uses $\phi$ to express his belief then the content of $\phi$ is identical to the content of his belief).

19 See Lewis (1983, p. 375; 1986, pp. 38–39). Lewis takes the ‘desire for a saucer of mud’ example from Anscombe (1957, §37).

20 The position defended in Sud (2019) can be interpreted in this way. Sud (2019) argues for a semantic account of vagueness, but his framework can be easily adopted to epistemicism.

21 This picture can be nicely mapped onto the inferentialist account presented by Peregrin (2014). Peregrin (2014, pp. 50–51) makes the distinction between an *inferential potential* and *inferential significance*; inferential potential is a broadly construed role that a sentence may play in inferences. Inferential significance is the role that a sentence plays in a given context. Thus, inferential potential can be treated as a function from circumstances to inferential significances. Our broadly-construed conceptual role of ‘*A*-ing is wrong’ can be understood as it’s inferential potential that has to be supplemented with idiosyncratic facts about the linguistic community to determine it’s inferential significance. Thanks to an anonymous reviewer for pointing out this connection.

22 That is not to say that the intensity of the negative emotion is the only dimension alongside which we can soritise ‘blame’. My goal is merely to show how the conceptual role in two communities can be very similar, but not identical (thus producing very similar, but distinct reference for moral terms).

23 One may be worried that there is a tension between the binary view e.g. on which we draw the boundary for ‘wrong’ at 300 s and assign a boundary of 200 s to another linguistic community with a different conceptual role. However, there is no tension. Suppose that our community uses ‘wrong’ with a conceptual role *R*_*@*_ (associated with the level of blame *B*_*@*_) and consequently the boundary of ‘wrong’ for looking away from one’s daughter is placed at 300 s; Community B has a conceptual role *R*_*B*_ (associated with the level of blame *B*_*B*_) and consequently the boundary is placed at 200 s. When we interpret Community B’s speech, we may hold simultaneously that:

- Given the conceptual role *R*_*B*_ that Community B employs, the property such that assigning it as reference to their use of ‘wrong’ would make them most reason-responsive is the one that places the boundary at 200 s AND
- Looking away for 200 s does not warrant blame.

For we may reason as follows. Since looking away for 300 s warrants the level of blame *B*_*@*_ and community B assigs level of blame *B*_*B*_ when using ‘wrong’ and the level of blame *B*_*@*_ is greater than *B*_*B*_, we should assign the Community B a lower cut-off point for ‘wrong’ than to ourselves (say at 200 s) as that would make them most reason-responsive (given the conceptual role ‘wrong’ plays in their community). We may still hold that Community would be more reason-responsive if they had employed the conceptual role for ‘wrong’ that we employ, but that is beside the point: given the conceptual role they employ, they get assigned a different cut-off than we do.

24 I believe that these issues are dealt decisively with in Hawthorne (2022).

25 See Dougherty (2014) and Constantinescu (2014).

26 See Barnes (2010), Barnes & Williams (2011), and Williams (2008) for thorough outlines of ontic vagueness.

27 See Williams (2017) for a survey of approaches to the problem.
